# Artificial Neural Networks Solve Musical Problems With Fourier Phase Spaces

**DOI:** 10.1038/s41598-020-64229-4

**Published:** 2020-04-28

**Authors:** Michael R. W. Dawson, Arturo Perez, Sara Sylvestre

**Affiliations:** Biological Computation Project, Department of Psychology, University of Alberta, Edmonton, Alberta Canada

**Keywords:** Computational models, Machine learning

## Abstract

How does the brain represent musical properties? Even with our growing understanding of the cognitive neuroscience of music, the answer to this question remains unclear. One method for conceiving possible representations is to use artificial neural networks, which can provide biologically plausible models of cognition. One could train networks to solve musical problems, and then study how these networks encode musical properties. However, researchers rarely examine network structure in detail because networks are difficult to interpret, and because many assume that networks capture informal or subsymbolic properties. Here we report very high correlations between network connection weights and discrete Fourier phase spaces used to represent musical sets. This is remarkable because there is no clear mathematical relationship between network learning rules and discrete Fourier analysis. That networks discover Fourier phase spaces indicates that these spaces have an important role to play outside of formal music theory. Finding phase spaces in networks raises the strong possibility that Fourier components are possible codes for musical cognition.

## Introduction

A main goal of studying musical cognition is identifying musical representations. Researchers seek musical representations by conducting psychological experiments^1–3^, or by using the methods of cognitive neuroscience^4–7^. However, the nature of musical representations is still unclear^7^.

A different method for generating new ideas about musical representations involves training artificial neural networks (ANNs). An ANN is a brain-like system of processors (analogous to neurons). Processors convert incoming signals into activity that is then sent to other processors via weighted connections (analogous to synapses). ANNs learn to convert stimuli into correct responses by modifying connection weights^8,9^. Many researchers train ANNs to solve musical problems^10–12^. In general, solving a musical problem requires discovering a mapping between representations of musical entities and classifications of these entities. For example, one musical problem would be classifying representations of different musical chords into chord types. While one could discover new musical representations by examining network structures to understand how they solve such classification problems^12^, this approach is rarely taken. It is widely assumed that ANNs are difficult to interpret because they capture subsymbolic properties^13–15^. Indeed, some music researchers are attracted to ANNs because they assume their networks capture important properties that *cannot* be formalized^13^.

Musical set theory provides a quite different, formal, approach to studying music^16–18^. Musical entities such as intervals, scales, or triads are combinations of pitch-classes that can be represented as musical sets. Musical set theory converts the twelve different Western pitch-classes (C, C#, D, D#, E, F, F#, G, G#, A, A#, B) into integers using the convention C = 0, C# = 1, and so on. For instance, using this scheme the C major triad (C, E and G) becomes the set (0, 4, 7). Mathematical operations on musical sets uncover striking similarities between musical entities that otherwise seem dissimilar^16^. Musical objects can also be encoded as ordered sets of twelve integers; the integer code of pitch-classes defines the order, while set members represent the number of each pitch-class present. Thus, the C major triad becomes (1, 0, 0, 0, 1, 0, 0, 1, 0, 0, 0, 0). Theorists find important musical regularities by decomposing such sets with the discrete Fourier transform (DFT)^19–23^.

Schuijer notes that critics worry that musical set theory is too formal to capture important informal characteristics^18^. If ANNs capture informal musical properties^13^, then we would expect that they are unrelated to musical set theory. Nevertheless, here we show striking relationships between networks and the discrete Fourier analysis of musical sets.

## An Example Network

**Figure 1:**
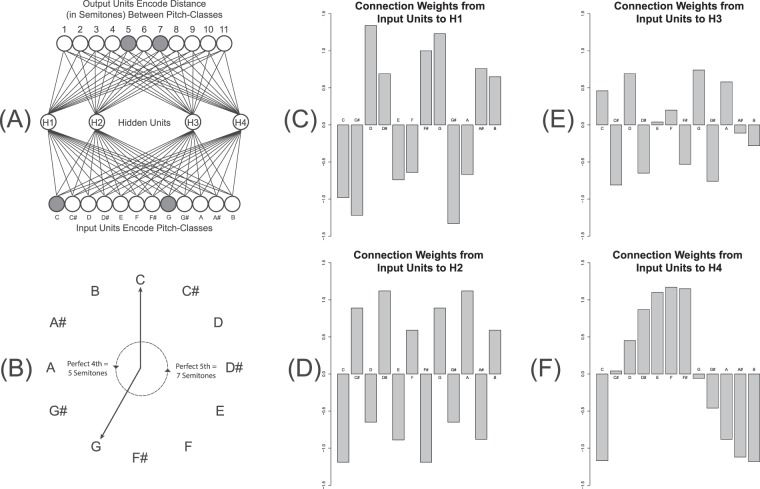
(**A**) An ANN for measuring distances between pairs of pitch-classes. Stimuli are presented by activating two input units; 1 A illustrates presenting C and G. The ANN learns to activate the output units that measure (as in (**B**)) the distances between the two pitch-classes by processing signals from four hidden units. All output and hidden units employ a Gaussian activation function *f(net) = e*^*−π(net-µ)2*^ that ranges between 0 and 1; *net* is the processor’s incoming signal, and *µ* is the function’s mean. Weights are modified by a variant of the generalized delta rule^9^. The bar plots (**C**-**F**) depict the weights of connections between input units and each hidden unit after training ends.

Figure 1A depicts an ANN for measuring the interval distance, in semitones, between pairs of pitch-classes. This task is equivalent to classifying pairs of pitch-classes into types of musical intervals. For instance, the pitch-classes C and G are separated by either 4 or 5 semitones depending on whether one measures distance clockwise or counterclockwise after arranging pitch-classes in a circle (Fig. 1B). The network is trained on 66 different pairs of pitch-classes using standard procedures^12^. To learn to respond correctly to each stimulus, the ANN requires four hidden units, each using a Gaussian activation function, to detect higher-order stimulus properties. Four hidden units is the minimum number that is required for the network to solve this problem. Previous research has shown that using the Gaussian activation function, and focusing on networks that are limited to the minimum number of hidden units required, increases the likelihood that the internal structure of a trained network can be understood^12,24^. A trained network’s connection weights from its input units to each hidden unit are plotted in Fig. 1C–F.

These connection weights show formal, but qualitative, musical properties^12^. For example, Processor H1’s weights (Fig. 1C) reveal *tritone balance*: two pitch-classes separated by a tritone (i.e. six semitones, such as C and F#, C# and G, etc.) are assigned weights equal in magnitude, but opposite in sign. Thus, the second six bars in Fig. 1C are produced by inverting the graph’s first six bars. Tritone balance is also true of H3 and H4 (Fig. 1E,F). H2 reveals *tritone equivalence*, in which pitch-classes a tritone apart are assigned the same connection weights (Fig. 1C). H2 also divides pitch-classes into two interval cycles in which adjacent members are separated by two semitones (e.g. once cycle is C, D, E, F#, G# and B). Members of one cycle are assigned a negative connection weight, while members of the other are assigned a positive weight.

**Figure 2:**
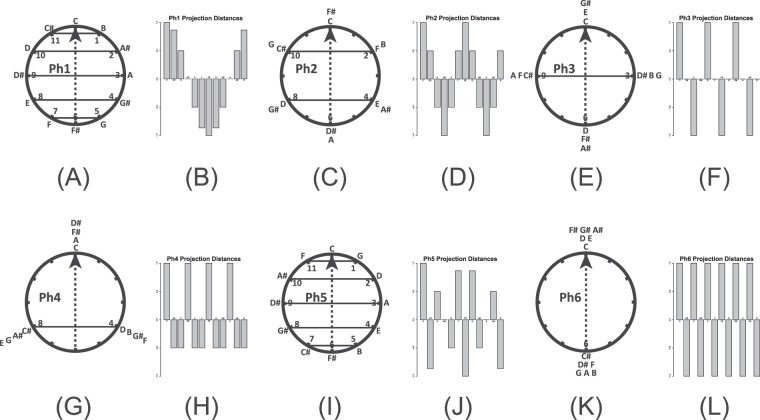
The six Fourier phase spaces, Ph1, Ph2, and so on used by Yust^23^. Each space represents the twelve pitch-classes on a clock face. (**A**) Phase space Ph1. The arrow, pointing at 0 o’clock, indicates a phase of 0. Solid lines denote projections from the pitch-class locations on the clock to the arrow. (**B**) Distances between each projection in (**A**) and the center of the phase space. Distances towards the arrowhead are positive. (**C**,**D**) Ph2 and its projection distances. (**E**,**F**) Ph3 and its projection distances. (**G**,**H**) Ph4 and its projection distances. (**I**,**J**) Ph5 and its projection distances. (**K**,**L**) Ph6 and its projection distances. The projection distance graphs show that each phase space represents a cosine function, and that the difference between phase spaces is the frequency of this function. Ph1 has a frequency of 1, Ph2 has a frequency of 2, and so on. Musical sets can be reconstructed by adjusting the phase of each component, weighting them and summing them together.

Crucially, Fig. 1C–F also suggest that weights are related to musical DFT components. Figure 2 presents the six Fourier phase spaces used by Yust to analyze musical sets^23^. Each phase space places the twelve pitch-classes at particular positions around a clock face whose center is (0, 0). If one plots the height (y-position) on the clock of each pitch-class in the order C, C#, D, and so on, the heights represent discrete samples of a cosine function. Phase spaces differ from one another with respect to cosine frequency. The first phase space has a frequency of 1, the second has a frequency of 2, and so on. Thus the phase spaces used by Yust provide harmonic components to be used to reconstruct a signal, where the signal in this case is a musical set.

Yust uses these phase spaces to perform a Fourier decomposition of a musical set as follows: For each phase space, vectors are drawn from the phase space origin to each pitch-class that is present in the set. These vectors are then added. The orientation of the resultant vector represents the phase of the phase space’s cosine function; the length of the resultant vector represents the magnitude of the cosine function. To generate Fourier components for reconstructing a musical set, an oriented arrow is drawn to bisect the phase space; the orientation is that of the resultant vector for that phase space. Yust then projects each pitch-class onto the arrow and measures the distance from the projection to the phase space’s origin (see the Fig. 2 bar graphs). When these projections are weighted by the magnitudes of the resultant vectors, the original musical set can be reconstructed.

Importantly, the qualitative structure of the phase space projections in Fig. 2 is similar to Fig. 1C–F. For example, tritone equivalence is true of the bar graphs for phase spaces Ph2, Ph4, and Ph6, while tritone balance is true of Ph1, Ph3, and Ph5. Ph6 also divides pitch-classes into two interval cycles based on cycles of major seconds.

**Figure 3:**
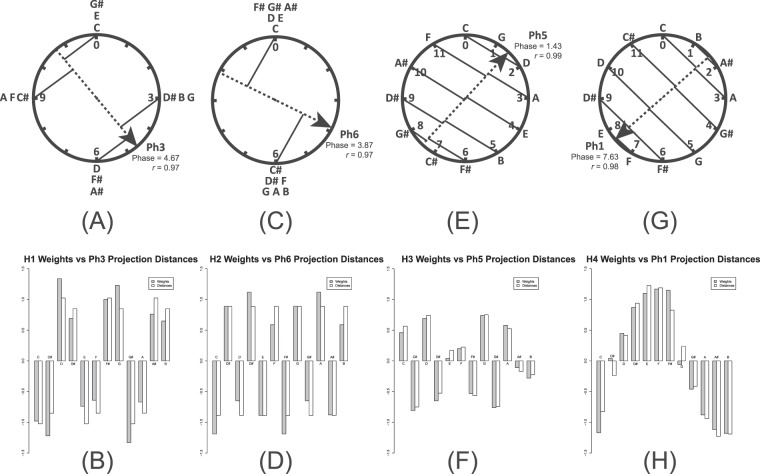
The results of fitting Fourier phase spaces to hidden unit weights for the Fig. 1 network. (**A**) The weights for processor H1 are best fit by Ph3 (see Fig. 2) with a phase of 2.47 (*r* = 0.97). (**B**) The relationship between H1 connection weights and projected distances after scaling the distances by 1.33. Projection distances are measured as described in Fig. 2. (**C**) H2 weights are best fit by Ph6 with a phase of 3.87 (*r* = 0.97). (**D**) H2 weights and projected distances scaled by 2.02. (**E**) H3 weights are best fit by Ph5 with a phase of 1.43 (*r* = 0.99). (**F**) The relationship between H3 weights and projected distances scaled by 0.774. (**G**) H4 weights are best fit by Ph1 with a phase of 7.63 (*r* = 0.98). (**H**) H4 weights and projected distances scaled by 1.25.

Is there a stronger, quantitative, relationship between hidden unit weights and Fourier phase spaces? To answer this question, we fit each set of hidden unit weights to each of six Fourier phase spaces. We rotated each phase space’s arrow to the position yielding the highest correlation between weights and projection distances. After finding the best-fitting phase, we used regression to predict weights from the distances in order to scale distances to match weights most closely. Figure 3 illustrates the results of this analysis for the Fig. 1 ANN; there is a high correlation between each set of weights and a single phase space, with *r* ranging from 0.97 to 0.99. Correlations between weights and the other phase spaces are much smaller.

The result described above is replicated by other networks that learn to solve one of three different musical problems as is detailed below.

## Methods and Materials

We trained networks to solve three different musical problems. In the first, 20 different ANNs learned to measure intervals (i.e. the task described above). Each network used four hidden units, and the training set consisted of 66 stimuli. In the second, 20 different ANNs learned to classify scales (a set of seven different pitch-classes) as belonging to one of seven different scale modes. Each network used five hidden units, and the training set consisted of 84 stimuli. In the third, 20 different ANNs learned to classify triplets of pitch-classes into four different triad types. Each network used three hidden units, and the training set consisted of 48 stimuli. For each problem, pilot simulations were used to determine the minimum number of hidden units required to solve the problem. The training sets used to train networks are provided as Supplementary Materials.

Standard procedures for training networks on musical problems were adopted^12^. Each network was trained using the generalized delta rule developed for processors that employ the Gaussian activation function^9^. This was accomplished using the Rumelhart software program available as freeware from the first author’s website (http://www.bcp.psych.ualberta.ca/~mike/Software/Rumelhart/index.html). Networks were trained epoch by epoch. During a single epoch of training, each training pattern was presented to the network once; the order of pattern presentation was randomized before each epoch. Connection weights were updated after each stimulus presentation. Prior to training, all connection weights in a network are initialized to random values between −0.1 and 0.1. The mean of each Gaussian activation function in a network was initialized to 0; these means were also modified by training. A learning rate of 0.005 was employed. Training proceeded until a network generated a ‘hit’ for every output unit for each pattern in a training set. A ‘hit’ is defined as activity of 0.9 or higher when the desired response is 1 or as activity of 0.1 or lower when the desired response is 0. Every network learned to solve its problem according to this criterion.

## Results

The networks trained in the three sets of simulations described above reproduced the general result reported earlier for the single network that was discussed in detail. That is, each set of connections weights has a very high correlation with one phase space, and much lower correlations with the other five. For the 80 different hidden units in networks trained to measure intervals (i.e. the 4 hidden units for each of the 20 networks trained), the average correlation between weights and the best-fitting phase space was 0.945 (S.D. = 0.056). For the 100 hidden units trained to classify scale modes (i.e. the 5 hidden units for each of the 20 networks trained), there was again a very high correlation between one phase space and a unit’s connection weights (mean correlation with best-fitting phase space = 0.881, S.D. = 0.131). For the 60 hidden units trained to classify triad types (i.e. the 3 hidden units for each of the 20 networks trained), each unit had a high correlation between their connection weights and a single phase space (mean correlation with best-fitting phase space = 0.92, S.D. = 0.08).

**Figure 4:**
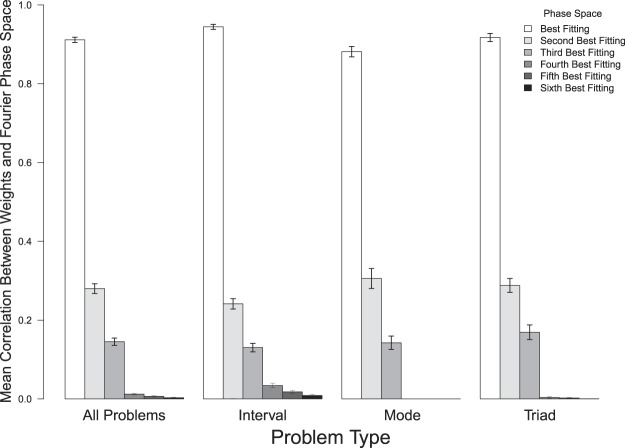
The mean correlations between hidden unit weights and Fourier phase space projections, with standard errors. The ‘All Problems’ data is based upon 240 different hidden units. The ‘Interval’, ‘Mode’, and ‘Triad’ data is based upon 80, 100, and 60 hidden units respectively. For each hidden unit, six different correlations were calculated, one for each phase space. The correlations were then ordered from the best fit to the worst fit, as is reflected in the six different bars presented for each problem type.

Figure 4 presents the mean correlation between connection weights and the six phase spaces for all of the simulations described above. For each condition, six different mean correlations were computed: the average correlation with the best fitting phase space, the average correlation with the next best fitting phase space, and so on. Figure 4 reveals that on average a hidden unit’s connection weights are highly related to a single Fourier phase space, and are far less related to the other five phase spaces. The average correlation between the weights and the best fitting phase space is striking higher than is the average correlation between the weights and the second best fitting phase space. That the standard errors of adjacent bars in Fig. 4 do not overlap indicates that the difference between them is statistically significant. Dependent t-tests confirm this result for all comparisons. For example, when all problems are combined the mean correlation for the best fitting phase space (0.91) is significantly higher than the mean correlation for the next best fitting phase space (mean = 0.28; *t* = 33.819, DF = 239, *p* < 2.2e-16). Similarly, the means for the same two bars for mode network problems (means of 0.88 vs 0.31) are significantly different (*t* = 15.123, DF = 99, *p* < 2.2e-16).

## Discussion

How do ANNs solve musical problems with the phase spaces they discover? Networks create a hidden unit space that codes each stimulus as a point whose coordinates are the hidden unit activities that it causes^12^. Gaussian output units carve this hidden unit space into decision regions with two parallel, narrowly separated, planes. To turn an output unit on, a point must lie between its planes. The network learns to position similar stimuli between the same planes, permitting an output unit to respond to them (and not to others). In music theory, Fourier components represent rich properties of musical objects, such as the complete set of musical intervals spanned by a set of pitch-classes^19–23^. Hidden unit activity represents the similarity between a phase space and a stimulus. Thus, when musical stimuli are arranged in hidden unit space, their positions are determined by their intervallic structure, which is explicitly encoded by Fourier phase spaces: the hidden unit weights.

Because our networks use Fourier components to represent intervallic structure, one might ask why we so not simply process our inputs with the DFT, bypassing the network altogether. However, the purpose of our research is to discover potential representations for musical cognition. Music theorists have already established the usefulness of analyzing musical sets with the DFT^19,21–23^. Nevertheless, establishing this usefulness is independent of exploring the kinds of representations that can be discovered by networks, and which in turn can become candidate cognitive representations. Our approach to discovering candidate representations *requires* using ANNs.

Furthermore, that ANNs discover discrete Fourier phase spaces is surprising because there is no obvious mathematical relationship between network training and computing the DFT. For instance, the DFT of a musical set requires correlating it with a various cosine and sine functions of different frequencies^19^. In contrast, ANNs are modified by small, iterative weight changes designed to reduce network response error^8,9^. Furthermore, the DFT is computed for individual musical entities. In contrast, our ANNs are trained with multiple stimuli; the phase spaces they discover are applied to *every* stimulus.

Nevertheless, that ANNs discover discrete Fourier phase spaces raises additional questions. Do processors that use a different activation function, like the logistic equation, also discover Fourier phase spaces? Are there certain musical problems that cannot be solved with this representation? Does the emergence of this sort of representation depend on how stimuli are encoded for networks? We are currently investigating these questions.

The discovery of discrete Fourier phase spaces in musical ANNs emphasizes the importance of conducting detailed examinations of networks^12^. It also reveals that these spaces have an important role to play beyond formal music theory. ANNs can generate new, biologically plausible, proposals about mental representation^14,25–27^. If basic learning rules for ANNs discover Fourier phase spaces, then this strongly suggests that such spaces are possible representations for musical cognition. Finding these representations in ANNs raises the possibility that Fourier phase spaces can bridge the cognitive psychology of music with its cognitive neuroscience^28^.

## Supplementary information

Supplementary Information.

## References

[1] Deutsch, D. *The Psychology Of Music*. 2nd edn, (Academic Press, 1999).

[2] Krumhansl, C. L. *Cognitive Foundations Of Musical Pitch*. (Oxford University Press, 1990).

[3] Sloboda, J. A. *The Musical Mind: The Cognitive Psychology Of Music*. (Oxford University Press, 1985).

[4] Abbott A (2002). Neurobiology: Music, maestro, please!. Nature.

[5] Peretz, I. & Zatorre, R. J. *The Cognitive Neuroscience Of Music*. (Oxford University Press, 2003).

[6] Peretz I, Zatorre RJ (2005). Brain organization for music processing. Annual Review of Psychology.

[7] Zatorre R, McGill J (2005). Music, the food of neuroscience?. Nature.

[8] Rumelhart DE, Hinton GE, Williams RJ (1986). Learning representations by back-propagating errors. Nature.

[9] Dawson MRW, Schopflocher DP (1992). Modifying the generalized delta rule to train networks of nonmonotonic processors for pattern classification. Connection Science.

[10] Griffith, N. & Todd, P. M. *Musical Networks: Parallel Distributed Perception And Performace*. (MIT Press, 1999).

[11] Todd, P. M. & Loy, D. G. *Music And Connectionism*. (MIT Press, 1991).

[12] Dawson, M. R. W. *Connectionist Representations of Tonal Music: Discovering Musical Patterns By Interpreting Artificial Neural Networks*. (Athabasca University Press, 2018).

[13] Bharucha, J. J. In *The Psychology Of Music* (ed. D. Deutsch) 413–440 (Academic Press, 1999).

[14] Smolensky P (1988). On the proper treatment of connectionism. Behavioral and Brain Sciences.

[15] McCloskey M (1991). Networks and theories: The place of connectionism in cognitive science. Psychological science.

[16] Forte, A. *The Structure Of Atonal Music*. (Yale University Press, 1973).

[17] Lewin, D. *Generalized Musical Intervals And Transformations*. (Oxford University Press, 2007).

[18] Schuijer, M. *Analyzing atonal music: Pitch-class set theory and its contexts*. (University of Rochester Press, 2008).

[19] Amiot, E. *Music Through Fourier Space: Discrete Fourier Transform in Music Theory*. (Springer International Publishing: Imprint: Springer, 2016).

[20] Callender C (2007). Continuous harmonic spaces. Journal of Music Theory.

[21] Quinn I (2006). General equal-tempered harmony: Introduction and Part 1. Perspectives of New Music.

[22] Quinn I (2007). General equal-tempered harmony: Parts 2 and 3 Perspectives of New Music.

[23] Yust J (2016). Special collections: Renewing set theory. Journal of Music Theory.

[24] Dawson, M. R. W. In *Cognition, Computation, and Pylyshyn* (eds. D. Dedrick & L. Trick) 175–199 (MIT Press, 2009).

[25] Dawson, M. R. W. *Minds And Machines: Connectionism And Psychological Modeling*. (Blackwell Pub., 2004).

[26] Rumelhart, D. E. & McClelland, J. L. *Parallel Distributed Processing, V.1*. (MIT Press, 1986).

[27] Bechtel, W. & Abrahamsen, A. A. *Connectionism And The Mind: Parallel Processing, Dynamics, And Evolution In Networks*. 2nd edn, (Blackwell, 2002).

[28] Tillmann, B., Bharucha, J. & Bigand, E. In *The Cognitive Neuroscience of Music* (eds. I. Peretz & R. Zatorre) 109–123 (Oxford University Press, 2003).

