# Supplementary material for: Artificial Neural Networks Solve Musical Problems With Fourier Phase Spaces
Source: Sci Rep. 2020 Apr 28;10:7151. doi: 10.1038/s41598-020-64229-4 (PMC7188679; doi:10.1038/s41598-020-64229-4)
Supplement: Supplementary file 1 — Supplementary Information. [file 41598_2020_64229_MOESM1_ESM.pdf]

Supplementary Materials for

Artificial Neural Networks Solve Musical Problems With Fourier Phase Spaces

Michael R.W. Dawson[1]*, Arturo Perez[1], Sara Sylvestre[1].

Correspondence to: mdawson@ualberta.ca

An online version of this document is available:
http://www.bcp.psych.ualberta.ca/MusicDFT/NatureSupplement.pdf

Materials and Methods

Software

All of the networks described in the paper were trained with the Rumelhart program for multilayer perceptrons.(*1*-*3*) This program is available as freeware from the first author's website: http://www.bcp.psych.ualberta.ca/~mike/Software/ .

Training Sets

The paper describes networks trained on three different tasks. The interval classification network is trained using the file Interval66.net (http://www.bcp.psych.ualberta.ca/MusicDFT/Interval66.net). The scale mode classification network is trained using the file ScaleMode.net (http://www.bcp.psych.ualberta.ca/MusicDFT/ScaleMode.net). The triad classification network is trained with the file Triads4Types.net (http://www.bcp.psych.ualberta.ca/MusicDFT/Triads4Types.net).

Each .net file is a text file. The first four lines dictate the structure of the network to be trained. This is followed by a sequence of rows that provide each of the training stimuli. The stimuli are followed by a sequence of rows that provide the desired output values for each stimulus. The structure of .net files is described in more detail elsewhere (*2*).

All three .net files define a network that uses 12 input units to encode the presence of pitch-classes. For the interval problem and the triad problem, input units are coded with 1 (indicating a pitch-class is present in a stimulus) or 0 (indicating the absence of a pitch-class). For the scale mode problem, pitch-class absence is coded as 0. However, pitch-class presence is coded as a fraction in order to represent the order of pitch-classes in a scale. The first pitch-class in a scale has its input unit activity coded as 1/7, the second as 2/7, and so on until the last pitch-class coded as 7/7.

Training Procedure

The training of the musical networks described in the paper follows the general procedures described in previous research (*11*). For any of the three problems, training proceeds by launching the Rumelhart program. The procedural description below assumes the use of a

Windows machine and the use of the Visual Basic version of the Rumelhart program. A beta-version of a Java Rumelhart program is available as freeware via a request to the communicating author. The Java version does not interface with Excel, but instead saves different files (one for weights, one for responses, etc.) in CSV format. These files can be opened using a spreadsheet program like Excel.

The program is then directed to load one of the training sets described earlier. Once loaded, the program opens a page for setting training parameters. To replicate the networks described in the paper, choose ‘All Value Units’ to use processors with the Gaussian activation function. Set the maximum number of epochs to 10,000. Set the learning rate to 0.005. For the scale mode problem and the triad classification problem, all other settings can be left at the default value. For the interval classification problem the values used to initialize connection weights also need to be altered. Choose ‘User Defined Starts For Weights’ and enter the value 0.25 in the top box (the box that displays 1.00). Then click ‘Use These Settings’.

Training proceeds by pressing the ‘Start Training’ button. Training proceeds epoch-by-epoch, where one epoch involves presenting each stimulus in the training set once. The order of pattern presentations is randomized before an epoch of training begins. Training proceeds until a ‘hit’ is recorded for each output unit and for each stimulus. Using the default settings, a ‘hit’ is defined as activity of 0.90 or higher when the desired activity is 1.00, or as activity of 0.10 or lower when the desired activity is 0.00. If the network does not converge after a large amount of training, then a local minimum has been encountered. One can train a new network by pressing the ‘Start Training’ button. If the network has not converged, but the network’s error is encouragingly small, additional training can be requested by pressing the ‘Continue Training’ button.

When training is over, one can examine the results by pressing the ‘Test Recall’ button. This provides a number of options; our standard practice is to double-click the option ‘Create A Summary In Excel’. This creates an Excel workbook that contains general information about the training, responses of the network to the training patterns, the connection weight structure of the network, and so on. Once network performance has been examined, one can select an option at the bottom of the screen to train the network from scratch, to continue to train the current network, or to load in a new problem for training.

An Excel spreadsheet is used to fit each of the discrete Fourier phase spaces to sets of hidden unit weights. This spreadsheet, WeightsToFourier.xlsm, is available as http://www.bcp.psych.ualberta.ca/MusicDFT/WeightsToFourier.xlsm. It uses a VBA macro to perform the fitting. The to-be-fit weights are pasted as columns onto the ‘Weights’ worksheet. (The spreadsheet comes loaded with the structure of the network whose analysis is reported in Figure 3.) One presses the button on this worksheet to fit the weights. A new worksheet will be created for each hidden unit that is fit, providing the best-fitting projections for each phase space, the phase for each of these spaces, and the correlation between weights and projected distances.

Results

The results of training each individual network are provided in the following location: http://www.bcp.psych.ualberta.ca/MusicDFT/TrainedNets/ . Each subdirectory in this folder holds the results for the three musical problems discussed in the manuscript. The results of training a single network are stored in a single Excel workbook. There are 20 different workbooks in each folder, one for each of the 20 networks trained on each problem.

The data used to create the bar chart in Figure 3 of the manuscript can be found online as Table S1: http://www.bcp.psych.ualberta.ca/MusicDFT/Tables/TableS1.pdf

The data that summarizes the best fit of each hidden unit to each of the six discrete Fourier phase spaces for the musical interval problem can be found online as Table S2: http://www.bcp.psych.ualberta.ca/MusicDFT/Tables/TableS2.pdf

The data that summarizes the best fit of each hidden unit to each of the six discrete Fourier phase spaces for the scale mode problem can be found online as Table S3: http://www.bcp.psych.ualberta.ca/MusicDFT/Tables/TableS3.pdf

The data that summarizes the best fit of each hidden unit to each of the six discrete Fourier phase spaces for the triad classification problem can be found online as Table S4: http://www.bcp.psych.ualberta.ca/MusicDFT/Tables/TableS4.pdf

References

1. M. R. W. Dawson, *Minds And Machines: Connectionism And Psychological Modeling*. (Blackwell Pub., Malden, MA, 2004).
2. M. R. W. Dawson, *Connectionism : A Hands-on Approach*. (Blackwell Pub., Oxford, UK ; Malden, MA, ed. 1st, 2005).
3. M. R. W. Dawson, *Connectionist Representations of Tonal Music: Discovering Musical Patterns By Interpreting Artificial Neural Networks*. (Athabasca University Press, Edmonton, AB, 2018).
